# Splice-altering variant in *COL11A1* as a cause of nonsyndromic hearing loss DFNA37

**DOI:** 10.1038/s41436-018-0285-0

**Published:** 2018-09-24

**Authors:** Kevin T. Booth, James W. Askew, Zohreh Talebizadeh, Patrick L. M. Huygen, James Eudy, Judith Kenyon, Denise Hoover, Michael S. Hildebrand, Katherine R. Smith, Melanie Bahlo, William J. Kimberling, Richard J. H. Smith, Hela Azaiez, Shelley D. Smith

**Affiliations:** 1 Molecular Otolaryngology and Renal Research Laboratories, Department of Otolaryngology, University of Iowa, Iowa City, IA USA; 2 Interdisciplinary Graduate Program in Molecular Medicine, Carver College of Medicine, University of Iowa, Iowa City, IA USA; 3 Developmental Neuroscience, Munroe Meyer Institute, University of Nebraska Medical Center, Omaha, NE USA; 4 Children’s Mercy Hospital and University of Missouri-Kansas City School of Medicine, Kansas City, MO USA; 5 Department of Otorhinolaryngology, Radboud University Nijmegen Medical Centre, Nijmegen, Netherlands; 6 DNA Microarray and Sequencing Core, University of Nebraska Medical Center, Omaha, NE USA; 7 Epilepsy Research Centre, Department of Medicine, University of Melbourne, Austin Health, Heidelberg, VIC Australia; 8 The Walter and Eliza Hall Institute of Medical Research, Parkville, VIC Australia; 9 Department of Medical Biology, The University of Melbourne, Parkville, VIC Australia

**Keywords:** *COL11A1*, DFNA37, nonsyndromic hearing loss, splice-site variant, exome sequencing

## Abstract

**Purpose:**

The aim of this study was to determine the genetic cause of autosomal dominant nonsyndromic hearing loss segregating in a multigenerational family.

**Methods:**

Clinical examination, genome-wide linkage analysis, and exome sequencing were carried out on the family.

**Results:**

Affected individuals presented with early-onset progressive mild hearing impairment with a fairly flat, gently downsloping or U-shaped audiogram configuration. Detailed clinical examination excluded any additional symptoms. Linkage analysis detected an interval on chromosome 1p21 with a logarithm of the odds (LOD) score of 8.29: designated locus DFNA37. Exome sequencing identified a novel canonical acceptor splice-site variant c.652-2A>C in the *COL11A1* gene within the DFNA37 locus. Genotyping of all 48 family members confirmed segregation of this variant with the deafness phenotype in the extended family. The c.652-2A>C variant is novel, highly conserved, and confirmed in vitro to alter RNA splicing.

**Conclusion:**

We have identified *COL11A1* as the gene responsible for deafness at the DFNA37 locus. Previously, *COL11A1* was solely associated with Marshall and Stickler syndromes. This study expands its phenotypic spectrum to include nonsyndromic deafness. The implications of this discovery are valuable in the clinical diagnosis, prognosis, and treatment of patients with *COL11A1* pathogenic variants.

## Introduction

Hereditary hearing loss is a genetically heterogeneous disorder with over 150 genes implicated. Mirroring the genetic complexity is the breadth of phenotypic manifestations associated with pathogenic variants in these genes as more than 20% exhibit an extraordinary pleiotropy: they can give rise to either autosomal dominant nonsyndromic hearing loss (ADNSHL) or autosomal recessive nonsyndromic hearing loss (ARNSHL) (e.g., *TECTA* and *TMC1*) and they can cause syndromic hearing loss or nonsyndromic hearing loss (NSHL) (e.g., Usher type 1–causing genes, *WFS1*, *TBC1D24*, and *COLL11A2*) (refs. ^1–6^).

The collagen family is diverse and consists of more than 20 genetically distinct genes. Collagens are fibrous structural proteins involved in the construction of skin, cartilage, bone, eye, and other tissues.^7,8^ All collagen molecules are comprised of three α-chain subunits tightly wrapped into a triple helix. The composition of each triple helix either contains one, two, or three different types of α-chains. For instance, the α-chains encoded by *COL11A1*, *COL11A2*, and *COL2A1* comprise a unique collagen fibril that is essential for proper skeletal and cartilage formation as well as ocular and auditory function.^6,9,10^

The *COL11A1* gene, located on chromosome 1p21.1, consists of 67 exons spanning 232 Kb.^11^ It encodes a peptide consisting of N- and C-terminal propeptides surrounding a collagen α-chain following the typical collagen Gly-X-Y repeat configuration. In the inner ear type XI collagens localize to the tectorial membrane, a gelatinous sheet-like structure anchored to the apex of the interdental cells. The tectorial membrane lies on top of sensory hair cells and is comprised of four distinct types of collagen (types II, V, IX, and XI) and three primary noncollagenous glycoproteins (α-tectorin; Tecta, β-tectorin; Tectb, and Otogelin).^12^

Pathogenic variants in *COL11A1* have been linked to specific genetic disorders of the connective tissue, namely Marshall syndrome (MRSHS),^13^ Stickler syndrome type II (STL2) (refs. ^14,15^), and fibrochondrogenesis (FBCG1) (ref. ^16^). Fibrochondrogenesis is an ultrarare disorder inherited in an autosomal recessive fashion. Affected individuals have severe skeletal defects characterized by pear-shaped vertebral bodies and broad long-bone metaphyses. Both Marshall and Stickler type 2 syndromes are rare autosomal dominant disorders. Clinically, there is much overlap between the two disorders because both include less severe vertebral and long-bone abnormalities; midface hypoplasia, which may include cleft palate; myopia with beaded vitreous; and mild to moderate hearing loss.

Until now, pathogenic variants in *COL11A1* have been exclusively linked to syndromic deafness. In this study, we present a novel splice-site altering variant in *COL11A1* that segregates in a large family with postlingual progressive ADNSHL and thus expands the phenotypic spectrum of pathogenic variants in *COL11A1*.

## Materials and methods

### Patients and clinical data

A four-generation family of European descent was ascertained as part of a genetic study of dominant progressive hearing loss at Boys Town National Research Hospital (BTNRH) between the years 1990 and 2000. After obtaining written informed consent from all participants with approval by the Institutional Review Board of BTNRH, pure tone audiograms and medical information were collected from participating family members. Clinical examination of the subjects excluded any additional syndromic findings. Blood samples from 48 family members were obtained and initial linkage studies were performed. The current studies were approved by the institutional review boards at the University of Nebraska Medical Center (UNMC) and the University of Iowa.

### Audiograms and data analysis

Pure tone audiometry was performed according to current standards to determine air conduction thresholds at 0.25, 0.5, 1, 2, 4, 6, and 8 kHz. Bone conduction thresholds were determined at some frequencies in some patients to exclude conductive hearing impairment. After validating binaural symmetry, the binaural mean air conduction threshold (dB hearing level, HL) at each frequency was used for further analyses. Conduction loss was recorded for some individuals. For this reason the threshold data from individual IV:12 were excluded, as well as those obtained at age 6 years from individual III:17, and those obtained from the left ear in individual III:4.

Linear regression analyses of threshold on age were used to evaluate progression of hearing impairment at the separate audio frequencies. These analyses comprised both individual longitudinal data derived from serial audiograms, and overall, cross-sectional last-visit data.^17^ Progression was called significant if the 95% confidence interval (95% CI) for slope did not include zero at two or more frequencies (out of 6 or 7, which is significant at *p* < 0.05 according to binomial distribution statistics, with *p* = 0.025 and *q* = 0.975 for positive correlations). The same applies to the threshold intercept. Progression was expressed in dB per year, and designated annual threshold deterioration (ATD). It was checked that the cross-sectional regression data conformed to the individual longitudinal regression data. Following that check, the regression data bearing on the last-visit thresholds were used to derive age-related typical audiograms (ARTA), which show the expected thresholds for a number of decade steps in age.^17^

Cross-sectional linear regression analyses were repeated for plots of threshold –P50presby against age, where P50presby is the median presbyacusis predicted by the ISO 7029 norm^18^ according to each patient’s gender and the age at which the audiogram was obtained.

### Linkage analysis

Genome-wide linkage analysis was first conducted with microsatellite markers using an ABI Prism linkage mapping set and results were confirmed using the Affymetrix Xba chip (Affymetrix, Santa Clara, CA) with 50,000 single-nucleotide polymorphism (SNP) markers.^19^ Genotype calls were made with the BRLMM algorithm. Parametric multipoint linkage analysis was carried out using the Merlin program.

### Exome sequencing and bioinformatic analysis pipeline

**Fig. 1:**
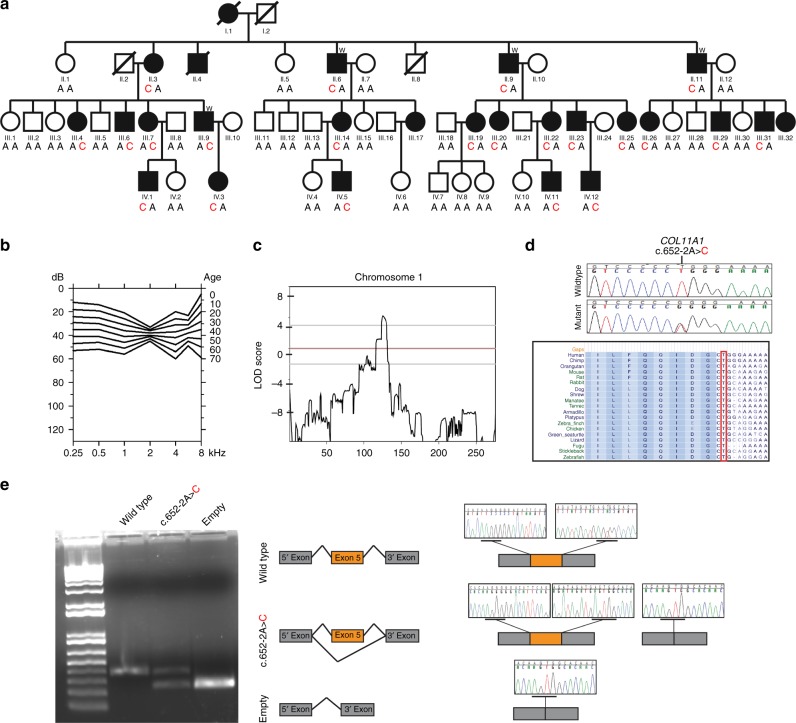
Genetic Analysis of c.652A>C variant in *COL11A1*. **a** Family pedigree showing the segregation of the c.652-2A>C variant in *COL11A1*. Red and bold indicates the mutant allele. Circles and squares represent females and males, respectively. Filled symbols denote individuals with nonsyndromic hearing loss (NSHL) and nonfilled symbols show individuals with normal hearing. **b** Age-related typical audiograms (ARTA). Binaural mean air conduction thresholds (dB HL) are presented for the ages 10–60 years. **c** Parametric linkage analysis plot of chromosome 1. **d** Representative chromatograms from wild-type and mutant sequences. **e** Gel electrophoresis of wild-type *COL11A1* exon 5, c.652-2A>C pathogenic variant, and the empty pET01 vector. The inclusion of exon 5 results in a 372-bp product and its exclusion results in a 234-bp band. Sequence chromatograms show read through at each exon junction. Results shown from COS7 experiments. *LOD* logarithm of the odds.

Four affected individuals (II.6, II.9, II.11, and III.9) underwent exome sequencing (ES) using the Agilent SureSelect Human All Exonv5 Kit (Agilent Technologies, Santa Clara, CA) as described^2,20^ (Fig. 1a). Prepared libraries were pooled and sequenced using an Illumina Hiseq 2000 (Illumina, San Diego, CA). We analyzed ES data using a custom bioinformatic pipeline. Reads were aligned to human reference genome (Human GRCh37/hg19) using Burrows–Wheeler Aligner (BWA). Variant calling was performed two ways: initially with the conservative Genome Analysis Toolkit (Broad Institute, Cambridge, MA), and later with the more inclusive SAMtools mpileup. Variants were annotated for conservation and deleteriousness using dbNSFP v2.0 (ref. ^21^), and for minor allele frequency (MAF) using the 1000 Genomes Project database, the Exome Aggregation Consortium database (ExAC) (http://exac.broadinstitute.org/), and the Genome Aggregation Database (gnomAD) (http://gnomad.broadinstitute.org/). Variant predicted effects on splicing were assessed using Human Splicing Finder (http://www.umd.be/HSF3/).

The following criteria were used for variant filtering: quality (depth ≥10×, Quality of Depth (QD) >5, quality >30), MAF <0.0001, coding effect (nonsynonymous, indels, and splice-site variants), heterozygosity, and allele sharing amongst sequenced affected individuals.

### Segregation analysis

Sanger sequencing was completed in available family members to confirm segregation of all candidate variants (Table S1); c.652-2A>C in *COL11A1* gene (MIM 120280; NM_080629.2), c.70C>T:p.R24W in *ARHGEF16* (NM_014448.3), and c.847G>A:p.E283K in *TRABD* (NM_001320484.1).

### Minigene splicing assay

In vitro minigene assays were carried out as described.^22^ Briefly, wild-type exon 5 and ~120 base pairs of each flanking intron of *COL11A1* were polymerase chain reaction (PCR) amplified and ligated into the pET01 vector (MoBiTec, Goettingen, Germany). The c.652-2A>C variant was introduced to the wild-type vector using the QuikChange Lightning Site-Directed Mutagenesis kit (Agilent, Santa Clara, CA, USA) according to the manufacturer’s protocol. Wild-type or mutant vectors were transfected into COS7 and HEK293 cells in triplicate. Total RNA was harvested 48 h posttransfection and complementary DNA (cDNA) was transcribed according to the manufacture protocol. PCR using primers specific to the 5′ and 3′ native exons of the pET01 vector was performed and products were visualized on an agarose gel. Gel products were extracted and Sanger sequenced.

## Results

### Clinical presentation

The family ascertained in this study is a four-generation kindred of European descent segregating hearing loss as an autosomal dominant trait (Fig. 1a). Pure tone audiometric evaluation of affected members showed bilateral, postlingual, progressive sensorineural hearing loss (Fig. 1b). The hearing loss was mild to moderate and progressed slowly. The finding of a significant threshold intercept at age 0 years (Figure S2) at all frequencies except 8 kHz (Figure S2) suggests the presence of a substantial congenital component (of 12 to 23 dB) to the SNHL. As shown by the ARTA depicted in Fig. 1b, the mean audiogram configuration developed from U-shaped (midfrequency type) to flat with advancing age up to ~40 years. At more advanced ages it remained flat or became very gently downsloping (Fig. 1b, S1).

To evaluate progression of hearing impairment at each frequency, we performed linear regression analyses of threshold on age. The resulting ATD (progression) was significant at 5 of 7 frequencies (significant): 0.25–1 kHz, 4 and 8 kHz. It varied between 0.2 and 0.8 dB per year (Figure S2). This variation is also reflected by the ARTA (Fig. 1b). One-way analysis of variance (ANOVA) followed by Tukey’s multiple comparison test showed that the ATD at 2 kHz was significantly smaller than the ATD at all other frequencies, whereas the ATD at 8 kHz was significantly greater than the ATD at all other frequencies, except for 0.25 and 4 kHz. The ATD for the thresholds corrected for median presbyacusis was significantly positive at 0.25–1 kHz (Figure S3). This suggests SNHL progression beyond presbyacusis at these lower frequencies. The ATD at the higher frequencies did not differ significantly from zero, even though the values at 6–8 kHz were negative. This implies that progression at 2–8 kHz conformed with expected presbyacusis. Clinical examination of affected members excluded any additional syndromic features usually associated with Marshall syndrome and Stickler syndrome as the craniofacial features of affected family members were normal. X-ray images of the long bones of the proband (III-19) were also normal. There was no history of ocular abnormalities or cleft palate in the family.

### Linkage analysis

The initial linkage analysis identified a single region on chromosome 1p21 spanning ~12 Mb between markers D1S497 and D1S2651 with a maximum LOD score of 8.29 for marker D1S195 (ref. ^19^). This linked interval was designated DFNA37. A second genome-wide linkage using the Affymetrix Xba chip narrowed down the linked interval to a ~8.4 Mb region between markers rs724480 and rs6667402 (Fig. 1c, S4).

### Exome sequencing and variant assessment

Capitalizing on the advances made in sequencing technologies, we used ES to screen four affected individuals (II.6, II.9, II.11, and III.9). An average depth of coverage of 114 reads was obtained with 92% of targeted regions covered at ≥30× (Table S2). After filtering for quality, MAF, coding effect (nonsynonymous, indels, and splice-site variants), heterozygosity, and allele sharing amongst sequenced affected individuals in the DFNA37 locus, only one variant in *COL11A1* was identified. The variant NM_080629.2; c.652-2A>C (chr1:103496802T>G) affects the canonical splice site in intron 4 resulting in the alteration of the acceptor site (AG to CG) confirmed by analysis with Human Splicing Finder 3.0 and NNSPLICE 0.9. Sanger sequencing performed on all family members showed the segregation of c.652-2A>C variant at a heterozygous state with the deafness phenotype in the extended family (Fig. 1a, d). This splice-site variant is predicted to cause skipping of exon 5 and a production of a protein lacking residues 218–260 in the N-propeptide domain.

### Splicing analysis

To characterize the impact of the c.652-2A>C variant on RNA splicing, we cloned the wild-type and mutant sequences of *COL11A1* exon 5 and flanking introns into the pET01 exon trap vector and transfected them into two different cell lines. Visualization of the splicing products revealed that cells transfected with the wild-type vector yielded the expected 372-bp band containing exon 5 (Fig. 1e). In contrast, cells transfected with the mutant vector yielded two bands; one at 372 bp corresponding to the wild-type allele and the second at 234 bp lacking exon 5. These results were identical across both cell lines. Sequencing all bands confirmed break points and splicing events.

## Discussion

Coupling linkage analysis and ES, we identified a novel splice-site altering variant (c.652-2A>C) in the *COL11A1* gene segregating in a large European-American pedigree with postlingual progressive ADNSHL. The DFNA37 locus was mapped almost two decades ago but screening methodologies at the time failed to detect causative alterations in *COL11A1* (ref. ^19^). Since mapping of DFNA37, knowledge of genomic sequences has improved substantially. In addition, development of next-generation sequencing has proven useful in elucidating the genetic causes of Mendelian disorders and notably, hereditary deafness.^23^ We capitalized on these new technologies to reevaluate the DFNA37 locus for pathogenic variants and identified the genetic cause underlying hearing loss in this family.

**Fig. 2:**
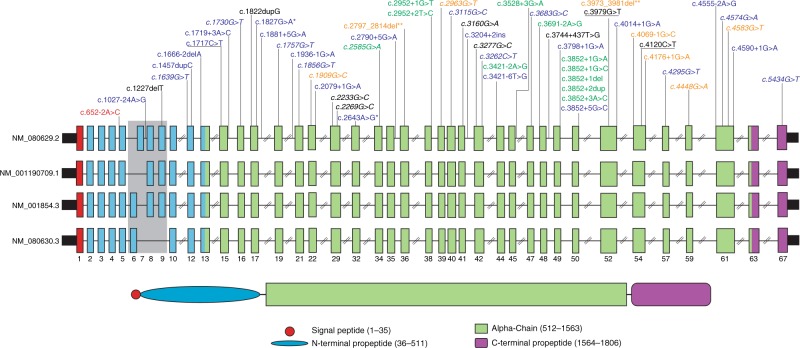
***COL11A1*** **gene and protein schematic denoting reported pathogenic variants and their associated phenotypes.** All variants were collected from the Deafness Variation Database (DVD; http://deafnessvariationdatabase.org/). The gray box shows the alternatively spliced exons. Text colored blue, black, green, and orange indicates the phenotypes associated with pathogenic variants in *COL11A1*: STL2, FBCG1, MRSHS, and STL2/MRSHS, respectively. Missense variants are in italics and nonsense variants are underlined. An asterisk (*) denotes a synonymous change, while a double asterisk (**) represents in-frame indels. The position of the c.652-2A>C pathogenic variant is shown in red and bold. Nucleotide numbering: the A of the ATG translation initiation site is noted as +1 using transcript NM_001854.3.

The heterozygous c.652-2A>C variant in *COL11A1* we identified segregating with the NSHL in this family is novel (absent from all population databases), highly conserved, and predicted to abolish the acceptor splice site of exon 5 by in silico analysis. We confirmed aberrant splicing of exon 5 due to c.652-2A>C in vitro using a minigene splicing assay (Fig. 1e). Interestingly, the c.652-2A>C variant seems to affect splicing efficiency rather than completely abolishing it. These findings suggest the c.652-2A>C variant creates a leaky acceptor splice site that allows for some expression of a normal spliced transcript. This might explain the audiometric variability seen among some affected individuals, such as the manifestation of conductive hearing loss seen in III.4 and IV.12 or the diversity of audiometric configurations between individuals which could be flat, gently downsloping or U-shaped (Figure S1). Exon 5 is the last exon before the start of the variable region (exons 6, 7, 8, and 9) thus the c.652-2A>C variant would affect splicing in all five transcripts of *COL11A1* (Fig. 2). Aberrant splicing leading to exon 5 skipping would result in an in-frame deletion and COL11A1 peptides lacking residues 218–260 in the N-terminal propeptide (Fig. 2). It is also possible that other splicing events occur that could not be detected with the current minigene design. Analysis of RNA from individuals harboring the c.652-2A>C variant would better define the splicing defects resulting from this variant.

The exact function of N-terminal propeptide remains unclear. However, it is known to have a role in regulating the shape and size of the collagen fiber.^24^ Lacking the amino acids encoded by exon 5 could result in a collagen fibril with a different diameter or shape, due to loss of key N-terminal propeptide regulatory sequences, such as the heparan sulfate binding motif that is located between residues 147–152 (refs. ^25,26^). Alternatively, it may result in protein misfolding as this domain also houses two structurally important cysteine residues at amino acids 236 and 243, which are important to disulfide bond formation with other cysteines at positions 182 and 61. In the inner ear, *COL11A1* is expressed in the tectorial membrane. Within the tectorial membrane, collagens form two networks of fibers: one unbranched, parallel, and coordinated mainly by noncollagenous proteoglycans. The second, a striated sheet structure, is orientated via the cross bridges and glycoproteins. Loss of this organization at either the collagenous or noncollagenous level causes hearing loss in mice and humans.^5,27^ Because the N-propeptide domain plays a role in the establishment of molecular interactions with several extracellular matrix molecules and cellular proteins such as heparan sulfate proteoglycans and calcium, its alteration might impair its binding affinity for these molecules.^25,28^ This could hamper interactions between cells and the surrounding extracellular matrix, as well as interactions between the diverse constituents of the extracellular matrix.

Regardless of the variant effect, it is clear the residues encoded by exon 5 are essential for proper auditory function. The absence of any other phenotypic manifestations in the described family is remarkable, given all previously reported autosomal dominant pathogenic variants (>50) have been only associated with either STL2 or MRSHS (Fig. 2). However, the splice-site variant identified in this study is the first pathogenic alteration reported in the nonvariable region of the N-propeptide domain. The majority of pathogenic variants in *COL11A1* are splice-altering located in the triple-helical domain and thought to exert their effect via a dominant–negative mechanism. This is further supported by studies in mouse showing that mice homozygous for a spontaneous frameshift pathogenic variant in *Col11a1* have severe chondrodysplasia and die at birth. Heterozygous mice escape lethality, develop osteoarthritis, and have normal auditory responses up to 10 months postnatally.^29^ The lack of an auditory phenotype in the heterozygous mouse suggests haploinsufficiency is not the pathogenic mechanism underlying *COL11A1*-related auditory defects in humans. Pleiotropy associated with deafness-causing genes, where pathogenic variants in the same gene could cause either syndromic or nonsyndromic hearing loss, has been demonstrated for several other genes such as the genes involved in Usher syndrome (*PCDH15*; DFNB23/USH1F, *USH1C*; DFNB18A/USH1C, *WHRN*; DFNB31/USH2D, *MYO7A*; DFNB2/DFNA11/USH1B, *CDH23*; DFNB12/USH1D), *WFS1* (DFNA6/14/38/Wolfram syndrome), *TBC1D24* (DFNA65/DFNB86/DOORS syndrome), and *COLL11A2* (DFNB53/DFNA13/STL3) (refs.^1–3,5^).

The present DFNA37 patients with a *COL11A1* pathogenic variant showed fairly similar audiograms to those reported for DFNA traits with a midfrequency type of hearing impairment: DFNA8/12 (*TECTA*) and DFNA13 (*COL11A2*) (refs. ^30,31^). The DFNB phenotypes that are allelic to these traits also show midfrequency-like types of audiograms, but usually at substantially higher thresholds: DFNB21 (*TECTA*) and DFNB53 (*COL11A2*) (refs. ^32,33^). Patients with collagenopathies that include hearing impairment due to deleterious variants in *COL11A1* (STL2 and Marshall syndrome) and COL11A2 (STL3) also show remarkably similar types of audiograms.^34–36^ The results of psychophysical tests at suprathreshold levels in DFNA8/12, DFNA13, and STL3 (*COL11A2*) patients revealed that the type of hearing impairment is compatible with intracochlear conduction loss.^36–38^ This is in line with the notion that disease-causing variants in *COL11A2* affect tectorial membrane function.^31,39^ Recent work on cochlear (micro)mechanics involving animal models with genetic modifications has provided compelling evidence about the relevant mechanical changes that can be involved.^5,12,39^

In summary, here we report the clinical and genetic characteristics associated with deafness at the DFNA37 locus and we expand the spectrum of *COL11A1*-associated phenotypes to include ADNSHL. This study illustrates another example of the pleiotropy exhibited by other deafness-causing genes and highlights the complexity associated with providing the correct genetic diagnosis.

## Electronic supplementary material

Supplementary Figure1

Supplementary Figure2

Supplementary Figure3

Supplementary Figure4

Supplementary Figure legend

Supplementary Tables
