# Supplementary figures and images for: Splice-altering variant in COL11A1 as a cause of nonsyndromic hearing loss DFNA37
Source: Genet Med. 2018 Sep 24;21(4):948–54. doi: 10.1038/s41436-018-0285-0 (PMC6431578; doi:10.1038/s41436-018-0285-0)

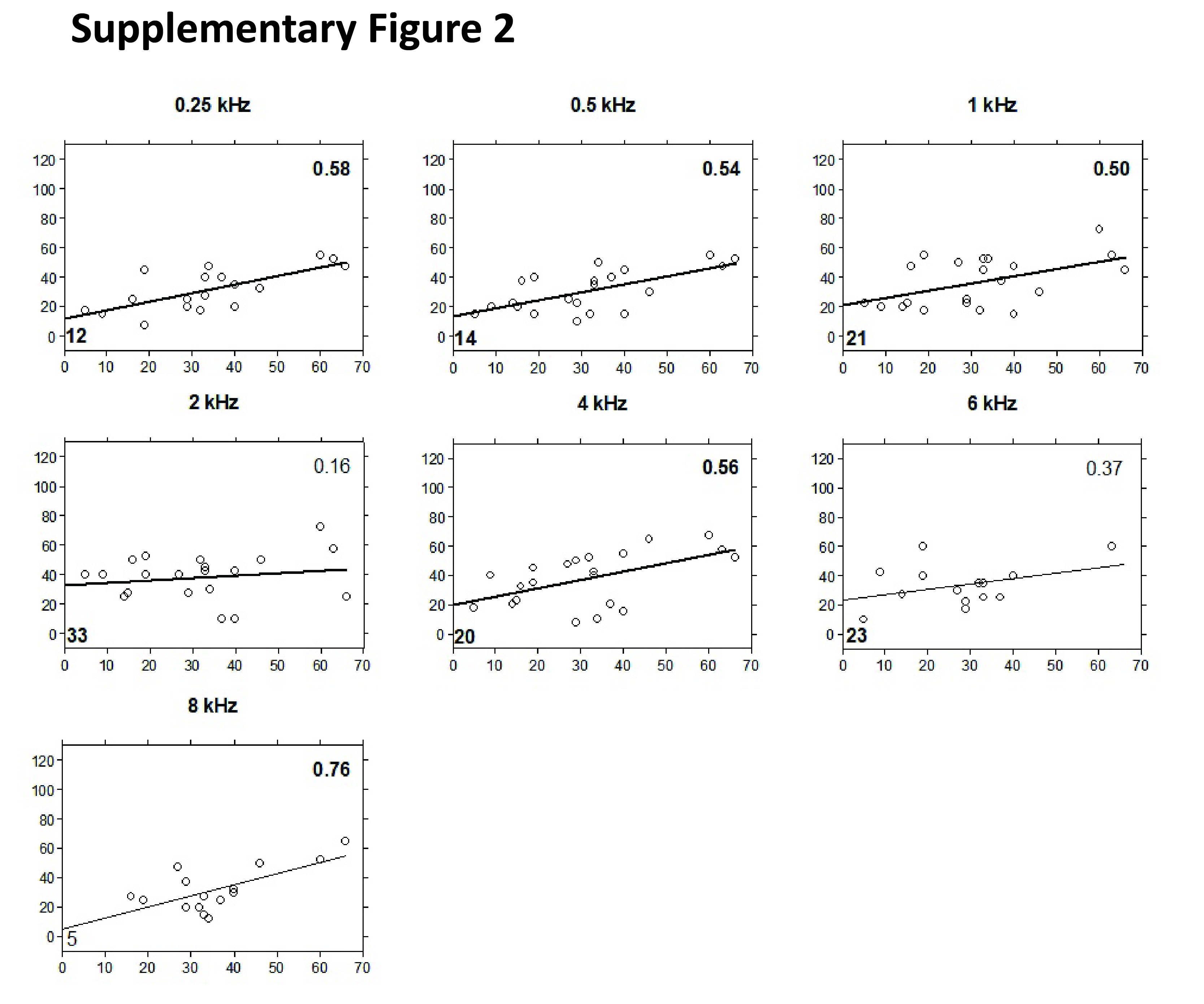

Supplement: Supplementary file 2 — Supplementary Figure2 [file 41436_2018_285_MOESM2_ESM.tif]

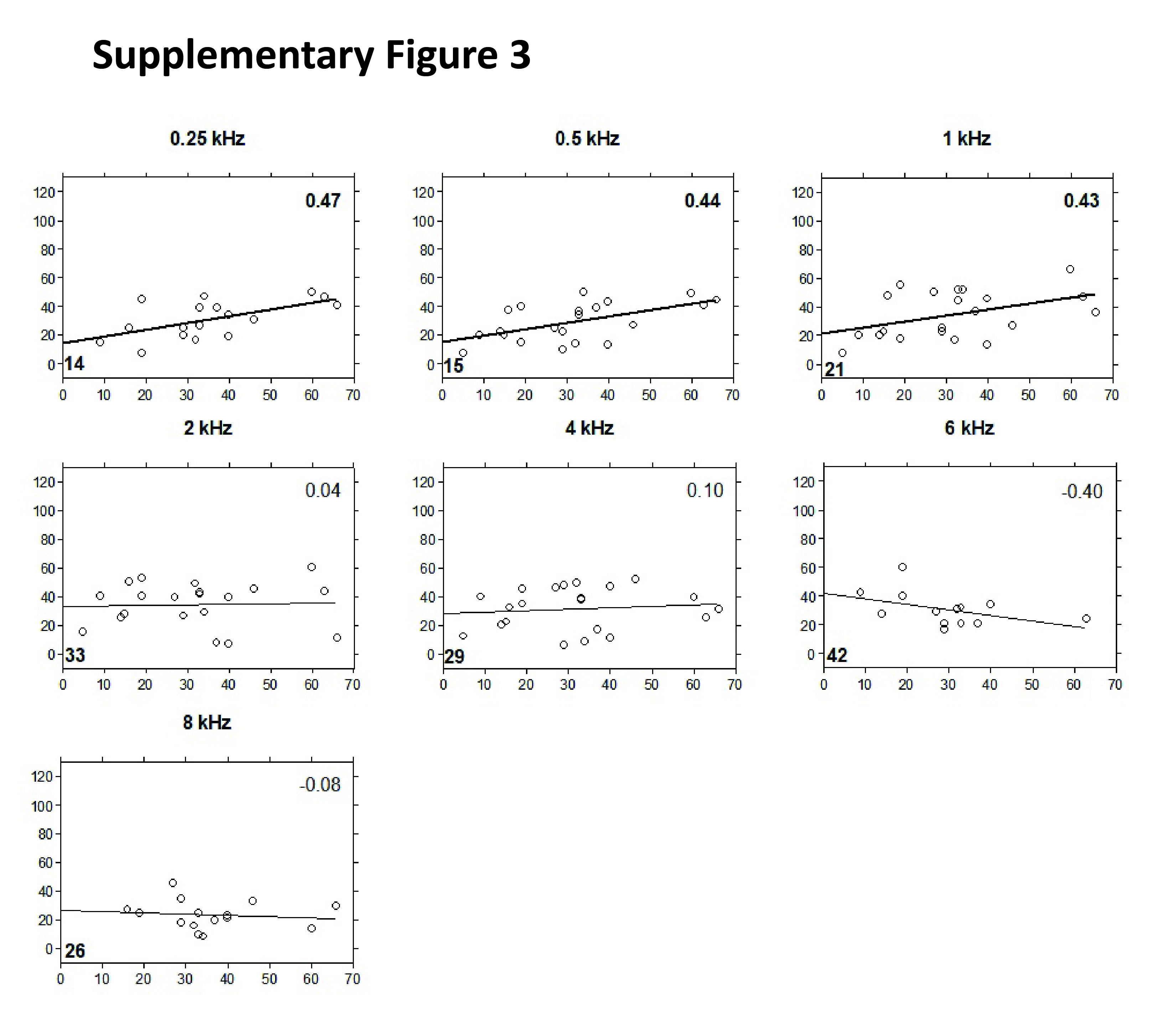

Supplement: Supplementary file 3 — Supplementary Figure3 [file 41436_2018_285_MOESM3_ESM.tif]

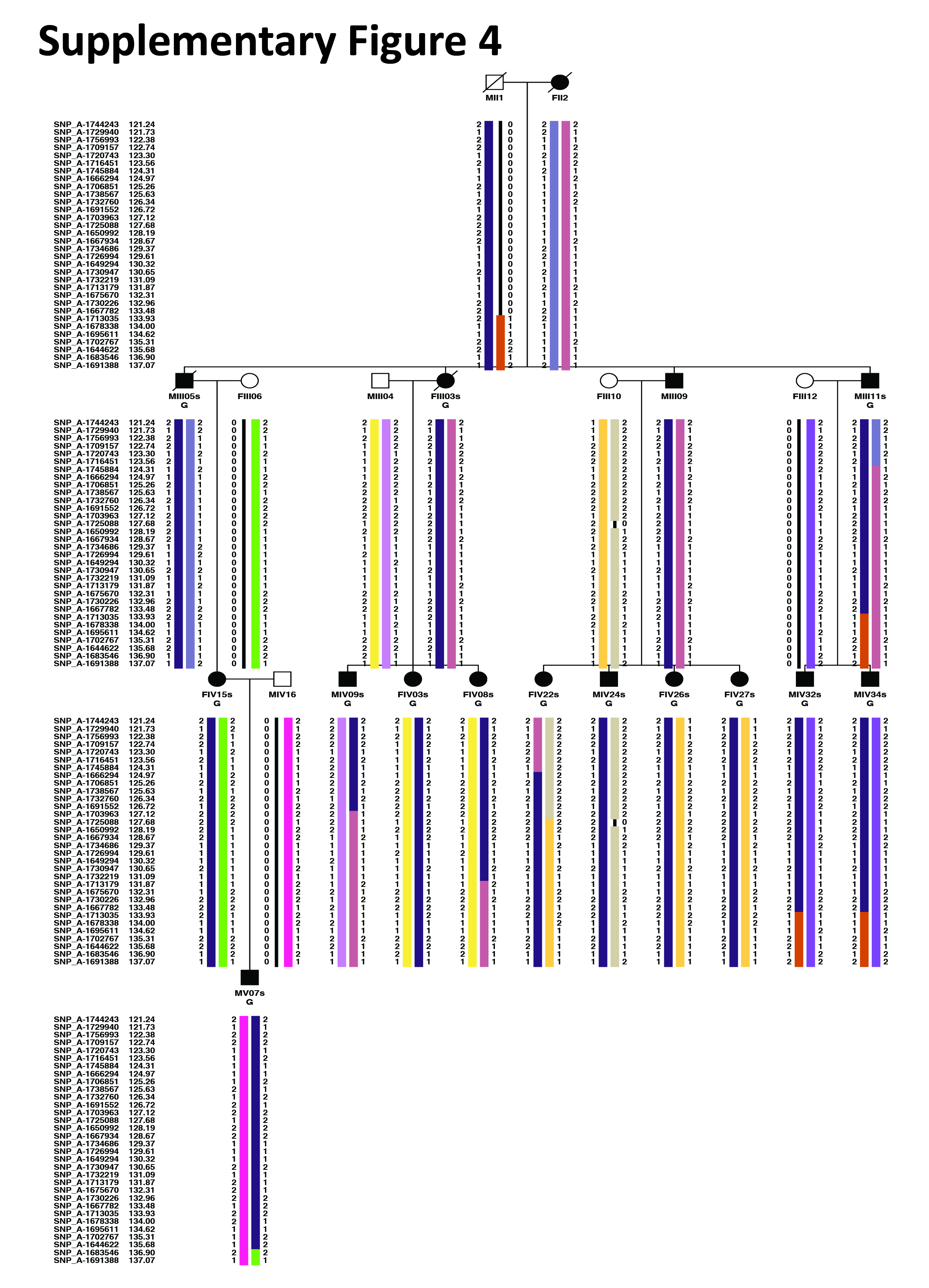

Supplement: Supplementary file 4 — Supplementary Figure4 [file 41436_2018_285_MOESM4_ESM.tif]
