# Supplementary material for: Splice-altering variant in COL11A1 as a cause of nonsyndromic hearing loss DFNA37
Source: Genet Med. 2018 Sep 24;21(4):948–54. doi: 10.1038/s41436-018-0285-0 (PMC6431578; doi:10.1038/s41436-018-0285-0)
Supplement: Supplementary file 5 — Supplementary Figure legend [file 41436_2018_285_MOESM5_ESM.docx]

**Supp Figure 1**

Patient audiometric data; panels ordered from top left to bottom right by the patient’s age (years) at the last visit. Audiograms were obtained using pure tone audiometry testing both air and bone conduction thresholds at octave frequencies from 250 to 8,000 Hz. The binaural mean air conduction threshold is shown. Individual IDs are shown above each panel, with F for female, M for male persons, and ns for obviously affected persons not screened for the mutation; R (person III:4) indicates monaural thresholds of right ear. There were no audiograms available for the mutation carriers III:20, IV:1, IV:3.

**Supp Figure 2**

Cross-sectional linear regression analysis of binaural mean threshold (dB HL) at the last visit (dB HL, open circles) on age (years). The regression line is plotted in each frequency panel, as a bold continuous line if progression is significant. The inset figures in the top-right and bottom-left corners of each panel are the annual threshold deterioration (ATD, dB/year) and the intercept (at age 0; dB HL), respectively, in bold when significantly different from zero.

**Supp Figure 3**

Cross-sectional analysis of these audiograms corrected for median presbyacusis. Positive slopes were found at the frequencies 0.25-4 kHz, significantly different (indicated by a bold regression line) from zero slope at 0.25-1 kHz, and negative slopes at 6-8 kHz.

**Supp Figure 4**

Refined linkage interval on chromosome 1. Affected individuals carry a morbid haplotype between markers rs724480 and rs6667402.
