# Supplementary material for: Splice-altering variant in COL11A1 as a cause of nonsyndromic hearing loss DFNA37
Source: Genet Med. 2018 Sep 24;21(4):948–54. doi: 10.1038/s41436-018-0285-0 (PMC6431578; doi:10.1038/s41436-018-0285-0)
Supplement: Supplementary file 6 — Supplementary Tables [file 41436_2018_285_MOESM6_ESM.docx]

Supplementary Table 1: Variant Summary

| **Gene** | **Genomic** | **HGVSc** | **HGVSp** | **GERP** | **phyloP** | **SIFT** | **PP2** | **LRT** | **MT** | **CADD** | **1KG** | **EVS** | **ExAC** | **GnomAD** | **MaxEnt Ref** | **MaxEnt Alt** | **MaxEnt Difference** |
| --- | --- | --- | --- | --- | --- | --- | --- | --- | --- | --- | --- | --- | --- | --- | --- | --- | --- |
| *COL11A1* | chr1:103496802:T>G | c.652-2A>C | - | C | C | - | - | - | - | 23.7 | 0 | 0 | 0 | 0 | 10.58 | 2.53 | 8.04 |
| *ARHGEF16* | chr1:3379718:C>T | c.70C>T | Arg24Trp | C | C | D | B | N | N | 26.0 | 0 | 0 | 0.026 | 0.01502 | - | - | - |
| *TRABD* | chr22:50636316:G>A | c.748G>A | Gly250Ser | C | C | - | - | D | D | 27.7 | 0 | 0 | 0.0015 | 0 | - | - | - |

Nucleotide numbering: the A of the ATG translation initiation site is noted as +1 using transcripts NM_080629.2, NM_014448.3, and NM_001320484.1 for *COL11A1, ARHGEF16* and *TRABD*, respectively. Minor allele frequencies are given in percentages. PP2, PholyPhen2; MT, MutationTaster; C, predicted conserved; D, predicted Damaging or Deleterious; B, Benign; N predicted neutral or polymorphism. An “-“ denotes no data available.

Supplementary Table 2: Coverage Statistics

| **Sample** | **II.6** | **II.9** | **II.11** | **III.9** | **Average** |
| --- | --- | --- | --- | --- | --- |
| **Total Reads** | 99681331 | 88126644 | 88739138 | 83694887 | 90060500 |
| **% Reads Overlapping Target** | 65.6% | 60.2% | 65.6% | 64.9% | 64.1% |
| **1X** | 99.3% | 99.1% | 99.3% | 99.3% | 99.3% |
| **10X** | 98.7% | 97.2% | 98.6% | 98.5% | 98.3% |
| **20X** | 97.3% | 93.3% | 96.9% | 96.3% | 96.0% |
| **30X** | 94.9% | 87.5% | 93.7% | 92.3% | 92.1% |
| **Average Depth of Coverage** | 128.8 | 105.1 | 114.8 | 107.3 | 114 |
